# Recent Changes in Drug Abuse Scenarios: The New/Novel Psychoactive Substances (NPS) Phenomenon

**DOI:** 10.3390/brainsci8120221

**Published:** 2018-12-13

**Authors:** Fabrizio Schifano

**Affiliations:** Psychopharmacology, Drug Misuse and Novel Psychoactive Substances Research Unit, University of Hertfordshire, Hertfordshire AL10 9AB, UK; f.schifano@herts.ac.uk

Over the last decade, the emergence of a vast range of new/novel/emerging psychoactive substances (NPS) has progressively changed drug market scenarios, which have shifted from the ‘street’ to a ‘virtual’/online environment.

Several definitions of NPS are in use, with the term ‘new’ not necessarily referring to new inventions but to substances that have recently been made available, possibly including failed pharmaceuticals or old patents which have been ‘rediscovered’ as ‘recreational’ molecules. Conversely, the term ‘novel’ can refer to something newly created, an old drug that has come back into fashion, or a known NPS molecule being used in an innovative or unusual way and hence presenting a ‘novelty’ appeal (Corkery et al., 2018) [1]. Though misleading, the terms ‘legal highs’ and ‘research chemicals’ have been used alternately to describe these molecules. NPS includes synthetic cannabinoids, cathinone derivatives, psychedelic phenethylamines, novel stimulants, synthetic opioids, tryptamine derivatives, phencyclidine-like dissociatives, piperazines, GABA-A/B receptor agonists, a range of prescribed medications, psychoactive plants/herbs, and a large series of image- and performance-enhancing drugs (IPED) (Schifano et al., 2015) [2]. Overall, users are typically attracted to NPS because of curiosity and the diffusion of social media users’ experiences, easy availability or affordability from online drug shops, legality, intense psychoactive effects, and the likely lack of detection in routine drug screenings (Schifano et al., 2015) [2].

Between 2004 and 2017, some 700–800 examples of NPS were reported by related European and international drug agencies (UNODC, 2018 [3]; EMCDDA, 2018 [4]), with most molecules identified being synthetic cannabinoids, synthetic cathinones, phenethylamine derivatives, and synthetic opioids. However, it could be argued that the NPS scenario is much larger than that outlined by those molecules which have been seized or formally identified by EU and international agencies. Since the online NPS scenario typically predicts the real life NPS scenario (Schifano et al., 2015) [2], identifying what is being discussed online by web-based NPS enthusiasts, or ‘e-psychonauts’ (Orsolini et al., 2015) [5], may well be of interest. With this in mind, a crawling/navigating software (i.e., the ‘NPS.Finder^®^’) was recently designed by our group. In November 2017, it started to automatically scan, on a 24/7 basis, a vast range of psychonaut web forums for NPS. After a year of operation, it has been possible to estimate that the online/psychonaut web forum NPS scene may include some 4000 different molecules. The most popular examples of NPS mentioned in psychonaut forums have included synthetic cannabimimetics, synthetic opioids, phenethylamines, designer benzodiazepines, and prescribed drugs.

NPS use, especially for synthetic cannabinoids and novel psychedelics, has been associated with a range of untoward medical consequences, including vomiting, seizures, cardiovascular complications, and kidney failure (Schifano et al., 2017) [6]. By contrast, the main focus of this special issue is on the major psychopathological consequences of NPS use. Indeed, due to their complex pharmacodynamics, there are increasing levels of concern about the onset of acute or chronic psychopathological issues associated with NPS intake.

The occurrence of psychosis has been related to: (a) increased central dopamine levels, typically seen with novel psychedelic phenethylamines, novel stimulants and synthetic cathinones; (b) significant cannabinoid CB_1_ receptor activation, which is associated with high potency synthetic cannabimimetics; (c) 5-HT_2A_ receptor activation, seen with latest generation phenethylamines, tryptamine derivatives and hallucinogenic plants; (d) antagonist activity at n-methyl-D-aspartate/NMDA receptors, observed with ketamine, methoxetamine/MXE, and their latest derivatives; and (e) k-opioid receptor activation, which is typically associated with both Salvia divinorum and Mitragyna speciosa/‘Kratom’ intake.

By considering the above, this special issue of Brain Sciences aims to provide an overview of a range of NPS-related issues. More precisely, Sahai et al. [7] present original *preclinical data* relating in silico and in vitro assessment of the psychoactive properties of a few dissociative diarylethylamines. Miolo et al. [8] focus on specific *analytical chemistry* issues relating to amphetamine-type stimulants and ketamine, while Parrott [9] argues that there are similarities between well-known recreational drugs and NPS in terms of *mood fluctuations/psychobiological instability issues*. Conversely, Cohen and Weinstein [10] present original *cognitive psychopharmacology* data relating to the use of organic and synthetic cannabinoids. From a *clinical point of view*, Bonaccorso et al. [11] introduce a case series of synthetic cannabinoid users presenting to acute psychiatric services with psychosis; Frisoni et al. [12] comment on the medical consequences of novel opioid intake; Martinotti et al. [13] provide a thorough overview of hallucinogen-persisting perceptual disorder, a clear issue of interest for NPS users; Schifano et al. [14] reflect on the misuse and abuse of prescribed medicines (e.g., benzodiazepine derivatives, methylphenidate look-alikes, and fentanyl analogues) in the NPS context; and Gittins et al. [15] provide empirical data relating NPS use by clients seeking treatment in the UK. Both Wadsworth et al. [16] and Miliano et al. [17] comment extensively on the *role of the open/deep web* in shaping and promoting changes in NPS scenarios. Finally, both Metastasio et al. [18] and Catalani et al. [19] offer original data which sheds further light on the expanding phenomenon of *IPED misuse/abuse*.

In conjunction with constant changes in basic structures from which emerging molecules can be derived, designed, and synthesized, the NPS market will continue to expand. This will pose a challenge, since NPS-related toxidromes are, per se, complex and unpredictable, and clinicians need to aim to be better educated in recognizing NPS-related toxicity issues. Drug control policies should be improved worldwide, and the list of examples of NPS should be constantly updated as improvements in analytical chemistry detection methods occur. Given the implications of NPS for mental health, psychiatric services should adapt to new drug scenarios while drafting new treatment strategies.

## References

[1] ReadCorkery J.M., Orsolini L., Papanti D., Schifano F., Miolo G., Stair J.L., Zloh M. (2018). Novel psychoactive substances (NPS) and recent scenarios: Epidemiological, anthropological and clinical pharmacological issues. Light in Forensic Science: Issues and Applications.

[2] Schifano F., Orsolini L., Duccio Papanti G., Corkery J.M. (2015). Novel psychoactive substances of interest for psychiatry. World Psychiatry.

[3] United Nations Office on Drugs and Crime (UNODC) (2018). World Drug Report 2018, Volume 3—Analysis of Drug Markets: Opiates, Cocaine, Cannabis, Synthetic Drugs.

[4] European Monitoring Centre for Drugs and Drug Addiction (EMCDDA) (2018). EMCDDA–Europol 2017 Annual Report on the Implementation of Council Decision 2005/387/JHA.

[5] Orsolini L., Papanti G.D., Francesconi G., Schifano F. (2015). Mind navigators of chemicals’ experimenters? A web-based description of e-psychonauts. Cyberpsychol. Behav. Soc. Netw..

[6] Schifano F., Orsolini L., Papanti D., Corkery J. (2017). NPS: Medical Consequences Associated with Their Intake. Curr. Top. Behav. Neurosci..

[7] Sahai M.A., Davidson C., Dutta N., Opacka-Juffry J. (2018). Mechanistic Insights into the Stimulant Properties of Novel Psychoactive Substances (NPS) and Their Discrimination by the Dopamine Transporter—In Silico and In Vitro Exploration of Dissociative Diarylethylamines. Brain Sci..

[8] Miolo G., Tucci M., Menilli L., Stocchero G., Vogliardi S., Scrivano S., Montisci M., Favretto D. (2018). A Study on Photostability of Amphetamines and Ketamine in Hair Irradiated under Artificial Sunlight. Brain Sci..

[9] Parrott A.C. (2018). Mood Fluctuation and Psychobiological Instability: The Same Core Functions Are Disrupted by Novel Psychoactive Substances and Established Recreational Drugs. Brain Sci..

[10] Cohen K., Weinstein A. (2018). The Effects of Cannabinoids on Executive Functions: Evidence from Cannabis and Synthetic Cannabinoids—A Systematic Review. Brain Sci..

[11] Bonaccorso S., Metastasio A., Ricciardi A., Stewart N., Jamal L., Rujully N.U., Theleritis C., Ferracuti S., Ducci G., Schifano F. (2018). Synthetic Cannabinoid use in a Case Series of Patients with Psychosis Presenting to Acute Psychiatric Settings: Clinical Presentation and Management Issues. Brain Sci..

[12] Frisoni P., Bacchio E., Bilel S., Talarico A., Gaudio R.M., Barbieri M., Neri M., Marti M. (2018). Novel Synthetic Opioids: The Pathologist’s Point of View. Brain Sci..

[13] Martinotti G., Santacroce R., Pettorruso M., Montemitro C., Spano M.C., Lorusso M., di Giannantonio M., Lerner A.G. (2018). Hallucinogen Persisting Perception Disorder: Etiology, Clinical Features, and Therapeutic Perspectives. Brain Sci..

[14] Schifano F., Chiappini S., Corkery J.M., Guirguis A. (2018). Abuse of Prescription Drugs in the Context of Novel Psychoactive Substances (NPS): A Systematic Review. Brain Sci..

[15] Gittins R., Guirguis A., Schifano F., Maidment I. (2018). Exploration of the Use of New Psychoactive Substances by Individuals in Treatment for Substance Misuse in the UK. Brain Sci..

[16] Wadsworth E., Drummond C., Deluca P. (2018). The Dynamic Environment of Crypto Markets: The Lifespan of New Psychoactive Substances (NPS) and Vendors Selling NPS. Brain Sci..

[17] Miliano C., Margiani G., Fattore L., De Luca M.A. (2018). Sales and Advertising Channels of New Psychoactive Substances (NPS): Internet, Social Networks, and Smartphone Apps. Brain Sci..

[18] Metastasio A., Negri A., Martinotti G., Corazza O. (2018). Transitioning Bodies. The Case of Self-Prescribing Sexual Hormones in Gender Affirmation in Individuals Attending Psychiatric Services. Brain Sci..

[19] Catalani V., Prilutskaya M., Al-Imam A., Marrinan S., Elgharably Y., Zloh M., Martinotti G., Chilcott R., Corazza O. (2018). Octodrine: New Questions and Challenges in Sport Supplements. Brain Sci..

